# A proper sequence of dynamic alignment in transtibial prosthesis: insight through socket reaction moments

**DOI:** 10.1038/s41598-023-27438-1

**Published:** 2023-01-10

**Authors:** Hiroshi Hashimoto, Toshiki Kobayashi, Fan Gao, Masataka Kataoka

**Affiliations:** 1 Osaka Metropolitan University, Habikino City, Osaka Japan; 2 Pacific Supply Co. Ltd., Daito City, Osaka Japan; 3 Department of Biomedical Engineering, Faculty of Engineering, The Hong Kong Polytechnic University, Hung Hom, Kowloon, Hong Kong China; 4 Department of Kinesiology and Health Promotion, University of Kentucky, Lexington, KY USA

**Keywords:** Biomedical engineering, Orthopaedics, Rehabilitation

## Abstract

Dynamic alignment in prosthetic fitting is important because it affects the user’s stability, kinematics, and kinetics such as socket reaction moments. It is performed by tuning the spatial relationship between the transtibial prosthetic socket and the foot following sequential observational gait analysis in the three anatomical planes. However, the order of planes in which the adjustment should be performed is still unclear. To investigate the appropriate sequence of dynamic alignment adjustment, ten participants with transtibial amputation were asked to walk in different alignment conditions (flexion, extension, adduction, abduction; lateral, medial, anterior, and posterior translation of the socket, and plantarflexion, dorsiflexion, inversion, and eversion of the foot) to measure socket reaction moments in the out-of-planes (e.g., the effect of sagittal alignment on the coronal moment). A significant difference was found only among socket posterior translation, socket flexion, and baseline alignment in the coronal moment (*P* = 0.02). The results of the current and previous studies suggest that moments in the coronal plane are affected by alignment changes in all three planes, whereas moments in the sagittal plane are affected only by sagittal alignment changes. It is suggested that the procedure of alignment adjustments should be finalized in the coronal plane.

## Introduction

The spatial relationship between the prosthetic socket and the prosthetic foot of a transtibial prosthesis is defined as “prosthetic alignment”^1^. Prosthetic alignment influences stability, comfort, spatiotemporal, kinetic, and kinematic parameters during gait in individuals with transtibial amputation^2–11^. Prosthetic alignment consists of angular and translational adjustments of the components in the prosthetic device. Angular adjustments include flexion, extension, adduction, and abduction of the socket, as well as dorsiflexion, plantarflexion, inversion, eversion, and internal/external rotation (toe-in/out) of the prosthetic foot. Translational adjustments involve anterior, posterior, medial, and lateral translation of the socket relative to the foot^1^. Traditionally, prosthetic alignment is tuned in the following sequence: bench alignment, static alignment, and dynamic alignment^12^.

Bench alignment is the alignment set on a workbench before fitting the prosthesis. It has been recommended as follows: the socket is flexed and adducted approximately five degrees, and the vertical line from the antero-posterior center of the socket at the mid-patellar tendon level falls anterior to the heel breast^12,13^ or 18–65 mm anterior to the center of the foot^14^ in the sagittal plane. In the coronal plane, it is recommended that the vertical line from the medio-lateral center of the socket falls 10–30 mm lateral to the center of the heel^12,13^. Pre-bench alignment assessment procedures have also been reported such as the vertical alignment axis method (VAA)^15–17^ and anatomically-based alignment (ABA)^14,16,17^. Nowadays, endoskeletal prostheses are predominant in the market and manufacturers have guidelines regarding bench alignment for each prosthetic foot.

Static alignment is performed while an individual with transtibial amputation is standing upright in their prosthesis. Prosthetists check the levelness of the pelvis to assess the height of the prosthesis, and examine whether the prosthetic foot is placed flat on the ground. The user is asked to comment on the comfort level and stability during fitting and standing with the prosthesis. When the position of the foot is inappropriate and/or the posture/balance is poor, the alignment needs to be adjusted^13^. Previous studies have suggested that the vertical component of the ground reaction force^18,19^ or the use of anteroposterior radiographs^20^ may be useful for establishing static alignment appropriately.

After the static alignment has been completed, the dynamic alignment is evaluated and adjusted based primarily on observational gait analysis. Visual gait observation and perception of users with prostheses are commonly used in clinical practice to guide dynamic alignment. The prosthetist first considers the user’s physical characteristics such as muscular strength, residual limb length, walking confidence and activity level. This is established using the observations by the prosthetists as well as feedback from the user of the prosthesis^12,13^. During the walking trials, prosthetists observe the gait of the individual with transtibial amputation to identify any kind of deviations in the sagittal, coronal and transverse planes. It should be noted that the gait deviations are generally considered to be linked with malalignment in the same plane as described in textbooks used for clinical education^1,12,13^. Prosthetists also ask the user for their comfort level when walking with the prosthesis. When a gait deviation is identified to be associated with malalignment, adjustments are made to minimize the deviation and/or discomfort, and then walking trials are performed again. This process is iterated until both the prosthetist and the user are satisfied^21^.

Previous studies have indicated that prosthetists’ judgement about the dynamic alignment may not be completely reliable and accurate. For example, feedback from prosthetic users such as their perception (e.g., comfort/complaint) about their prosthesis during walking is primarily expressed verbally, and the clinicians need to carefully interpret it and connect to potential contributing factors. It has also been reported that the perception of prosthetic users is not always accurate and does not necessarily reflect the prosthetic alignment changes^22,23^. Observation of gait deviation is focused on kinematic and temporo-spatial parameters. Though some joint angles were reported to be affected by alignment changes (e.g., increased internal rotation of prosthetic feet may increase maximum knee flexion angle)^24^, effects of alignment changes on kinematic parameters may not be predictable^25^. Similarly, temporo-spatial parameters may be influenced by alignment changes, but might not serve as a good predictor of prosthetic alignment changes^25^. These findings are consistent with a report by Zahedi et al., which showed that observational gait analysis of prosthetic alignment changes by prosthetists may not be reproducible^26^.

In contrast, kinetic parameters may be useful to evaluate deviations in prosthetic alignment. Ground reaction forces have been reported to be affected by prosthetic alignment changes during walking^5,7,10^. Moment of force measured with load cells displayed a strong correlation with intra-socket pressure^10^. Socket reaction moment, or external moment of force measured with an embedded load cell in prostheses, has been reported to be a good predictor to alignment changes of transtibial prostheses^27–29^. Chen et al. reported that the use of socket reaction moment would lead to a similar prosthetic alignment to conventional methods based on observation of prosthetists and feedback of the user although it resulted in slightly higher varus moments^30^.

**Figure 1:**
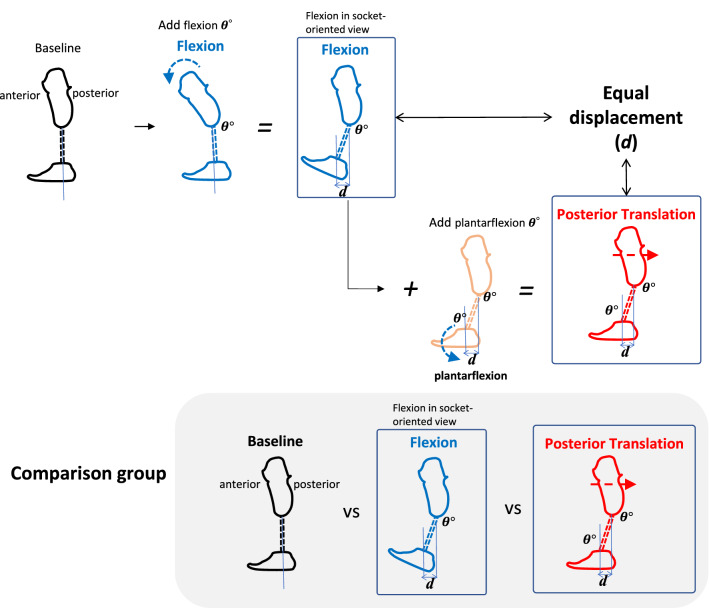
Equal displacement (*d*) of the prosthetic foot in angular changes and translational changes.

Dynamic alignment needs to be addressed across all three anatomical planes (sagittal, coronal, and transverse), however, an agreement about their sequence and/or priority for quick and accurate prosthetic alignment has not been consistently reached in rigorous peer-reviewed research. Several authors have suggested a specific sequence for adjustments across each plane, but there has been little agreement of this sequence. It was recommended by Radcliffe et al. that dynamic alignment should be first tuned in the coronal plane, followed by the sagittal plane, because stability in the sagittal plane is crucial and should not be achieved until adjustments in the coronal plane have been completed^12,13^. It has also been reported that linear (or translational) adjustment should be performed first, followed by tilt (or angular) adjustment^31^. However, it is still unclear whether these sequences (e.g., first in the coronal, then sagittal plane, or translation first, then angulation) are reasonable. It has been reported that transtibial alignment changes in the sagittal plane significantly affect the moments in the coronal plane, however, alignment changes in the coronal plane did not reciprocally influence the sagittal plane moments^32^. Based on these findings, Kobayashi et al.^32^ suggested that dynamic alignment should be conducted first in the sagittal plane followed by the coronal plane. Thus, the socket reaction moment may be useful to determine the order of adjustment in the dynamic alignment. Furthermore, it should be noted that the conclusion of their study appears inconsistent with Radcliffe et al.’s recommendation. Also, their study^32^ did not clarify the differences in the effects between translational and angular alignment changes on moments when the displacement of the foot from the socket is equal (*d* in Fig. 1)^23,33^. The difference of effects in out-of-plane moments between angular changes and translational changes should be investigated with equal displacement in order to consider the order of angular and translational adjustments. Toe-in/toe-out angles of prosthetic feet have also been reported to significantly affect moments in the out-of-plane (e.g., in the coronal plane)^29^. It also should be clarified whether angulation changes of prosthetic feet in the sagittal or coronal plane (i.e., dorsiflexion/plantarflexion, or inversion/eversion) influence moments in the out-of-planes. Examining the effect of alignment changes of the socket or the foot on the socket reaction moments in out-of-planes could reveal the appropriate sequence of dynamic alignment adjustment in the transtibial prosthesis.

The aim of this study was to investigate the effects of alignment changes of transtibial prostheses on the out-of-plane socket reaction moments in order to seek the appropriate sequence of dynamic alignment adjustment. We hypothesized that the sequence of planes in dynamic alignment could be determined based on the out-of-plane effects of the alignment changes on the socket reaction moments^29,32^.

## Methods

### Participants

Ten participants (nine males and one female) were recruited who were also the same participants group from a previous study^33^ (Table 1). Their mean (SD: standard deviation) age, height, and body mass were as follows: 51.2 (13.5) years old, 170 (7.8) cm, and 67.7 (8.5) kg. The inclusion criteria were having a unilateral transtibial amputation and being a community ambulator without walking aids. The exclusion criteria were having any orthopedic and/or neurological disorders and being under the age of twenty. This study was approved by the Internal Review Board of the Graduate School of Comprehensive Rehabilitation, Osaka Prefecture University (Approval number: 2018–101) and conducted according to the Declaration of Helsinki guidelines. Written informed consent was obtained from all participants.

**Table 1:** Demographic and anthropometric information on participants.

ID	1	2	3	4	5	6	7	8	9	10
Gender	m	m	m	m	m	f	m	m	m	m
Height (cm)	169	175	172	163	172	153	180	171	182	172
Body mass (kg)	58	65	82	57	65	83	73	64	63	67
Age	37	41	55	69	59	42	44	70	65	30
Length of residual limb (mm)	169	120	195	110	200	140	145	140	230	150
Period of prosthetic usage (year)	1	40	35	26	50	8	17	14	0.5	0.2
Height from floor to end of socket (mm)	220	295	170	225	220	190	240	225	182	202
Side of amputation	r	r	r	l	r	l	l	l	r	l
Cause of amputation	Trauma	Congenital	Trauma	Trauma	Tumor	Tumor	Trauma	Trauma	Diabetic	Trauma
Activity level (K-classification)	3	4	4	3	4	3	4	3	3	4
Prosthetic socket	TSB	PTB	TSB	TSB	TSB	TSB	TSB	TSB	TSB	TSB

Mean height (standard deviation): 170 (7.83) cm, body mass: 67.7 (8.52) kg, age: 51.2 (13.52) years old, length of residual limb: 159.9 (36.1) mm, period of prosthetic usage: 19.17 (16.96) years, height from floor to end of socket: 216.9 (33) mm. Abbreviations: f: female, m: male, l: left, r: right, TSB: total surface bearing, PTB: patella tendon bearing.

### Instruments

An instrumented prosthetic pyramid (Europa, Orthocare Innovations LLC, Edmonds,WA, USA) was used to measure the magnitude and timing of socket reaction moments^30,34,35^. A three-dimensional motion capture system (Vicon, Vicon Motion Systems Ltd., UK) with twelve infrared cameras and two force plates (AMTI, USA) was used with a Plug-in-Gait marker set^36^ to measure walking parameters including speed. The sampling rate was set at 100 Hz for the Europa and the motion capture system, and 1000 Hz for the force plates.

**Figure 2:**
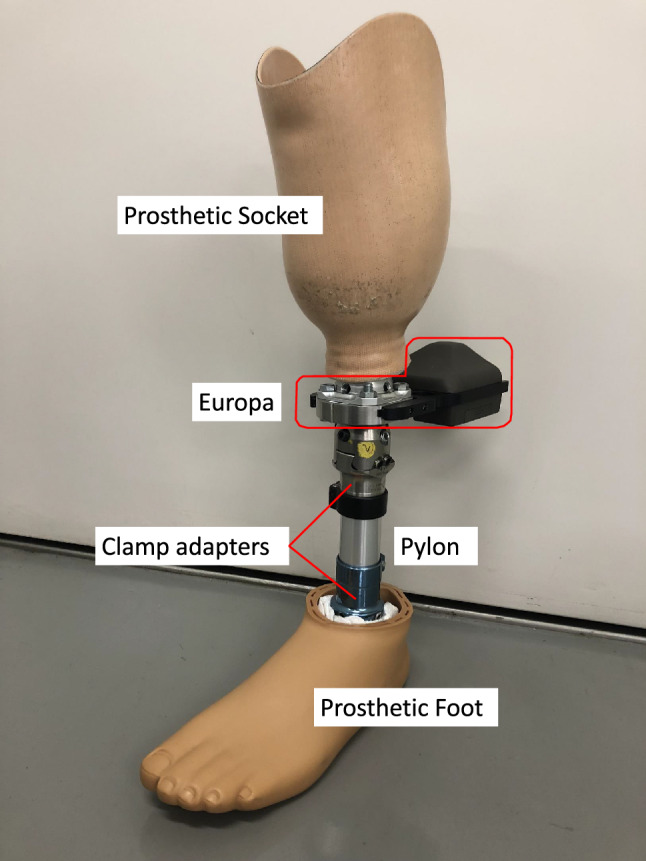
An experimental prosthesis with the instrumented prosthetic pyramid (Europa).

A clamp adapter, a slide adapter, a pylon, a prosthetic foot (LP Vari-Flex, Ossur HF, Reykjavik, Iceland), and the instrumented prosthetic pyramid were used to build an experimental prosthesis using the participant’s own socket (Fig. 2). ID2 used PTB socket with a cuff suspension strap, whereas other participants used TSB sockets with silicone locking liners for suspension. A digital level gauge (DP200Hi, STS Co. Ltd., Japan) was used to check the angular changes of each condition.

### Protocol

**Figure 3:**
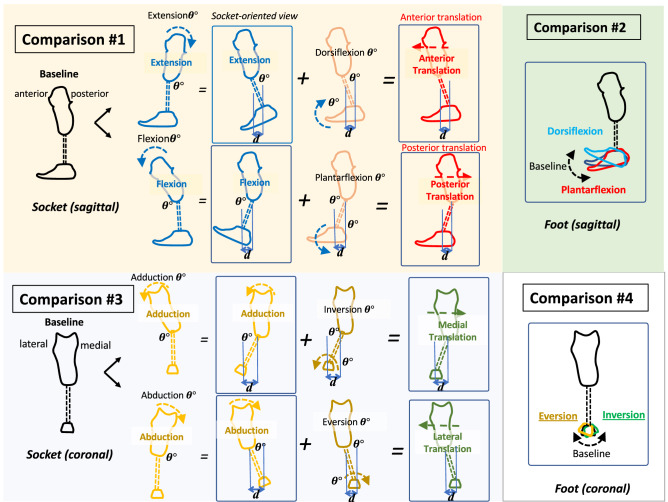
Comparison of alignment changes.

The instrumented prosthetic pyramid was installed at the bottom of each participant’s prosthetic socket. Baseline alignment was set up by a prosthetist based on observation, participants’ preferences, and the Compas system (Orthocare Innovations LLC, Edmonds,WA, USA)^30^. Alignment in the sagittal plane included the following conditions: (1) three-degree flexion/extension of the socket, anterior/posterior translation of the socket, (2) three-degree dorsiflexion/plantarflexion of the foot. Alignment conditions in the coronal plane included: (3) six-degree adduction/abduction of the socket, medial/lateral translation of the socket, (4) six-degree inversion/eversion of the foot. Adjustment in the sagittal plane was limited in order to compensate for the heel height of the prosthetic foot^33^. These conditions were set with respect to each individual’s baseline alignment. The translations were established with equal displacement with angular changes^33^ as shown in Figs. 1 and 3. The mean (standard deviation) of translational perturbations were 22.67 (3.49) mm in six-degree angular changes and 11.35 (1.75) mm in three-degree angular changes.

The participants were asked to walk on a 15-m walkway at a self-selected speed, and data were collected under each alignment condition. The intervention was one-side blinded: the conditions of alignment were set by the same prosthetist in randomized orders and the participants were not informed of the order of alignment conditions. All the participants were accustomed to walking without footwear due to their local culture, therefore shoes were not used and their potential influence on gait was eliminated. Participants were given sufficient time to acclimatize themselves to the 15-m walkway before each condition.

### Data processing and statistical analysis

To collect and export data of the socket reaction moments for data processing, Compas version 1.3.2 (Orthocare Innovations LLC, Edmonds,WA, USA) was used. In the sagittal plane, maximum flexion moments, % stance of maximum flexion moments, maximum extension moment, % stance of maximum extension moment, and zero-cross (the timing when the moments cross zero) were measured and averaged. In the coronal plane, moments of 5%, 20%, and 75% stance were measured and averaged. Negative values of coronal/sagittal socket reaction moments were defined as varus/flexion moments, respectively. The magnitudes of moments were normalized by participants’ body mass. These parameters were selected according to previous studies^23,29^.

The statistical analysis was performed in four comparison groups (Fig. 3) as follows:

*Comparison #1*: the effects of the socket alignment changes in the sagittal plane on the coronal socket reaction moments, including baseline-flexion-posterior translation and baseline-extension-anterior translation.

*Comparison #2*: the effects of the foot alignment changes in the sagittal plane on the coronal socket reaction moments, including baseline-plantarflexion-dorsiflexion.

*Comparison #3*: the effects of the socket alignment changes in the coronal plane on the sagittal socket reaction moments, including baseline-adduction-medial translation and baseline-abduction-lateral translation.

*Comparison #4*: the effects of the foot alignment changes in the coronal plane on the sagittal socket reaction moments, including baseline-inversion-eversion in the coronal plane.

Walking speeds in each alignment condition were also averaged and compared within these comparison groups.

When normal distribution was confirmed by Shapiro–Wilk tests, repeated measures analysis of variance (ANOVA) was performed, and otherwise, Friedman tests were performed, which was followed by post hoc tests with the Bonferroni method for multiple comparisons. Effect size was also calculated for each comparison (Partial eta squared for repeated measures ANOVA / Kendall’s W for Friedman tests).* P* < 0.05 was defined as significant and SPSS ver. 26 (IBM Corporation, USA) was used for the statistical analysis.

## Results

### Outcome of the normality test

According to the results of Shapiro–Wilk tests, repeated measures ANOVA were performed for all comparisons except for 20% stance of coronal socket reaction moment among baseline, posterior translation and flexion, and zero-cross and percent stance of peak extension moment among baseline, adduction, and medial translation, for which Friedman tests were performed.

### Effects of sagittal alignment changes of the socket on the coronal socket reaction moments (comparison #1)

**Figure 4:**
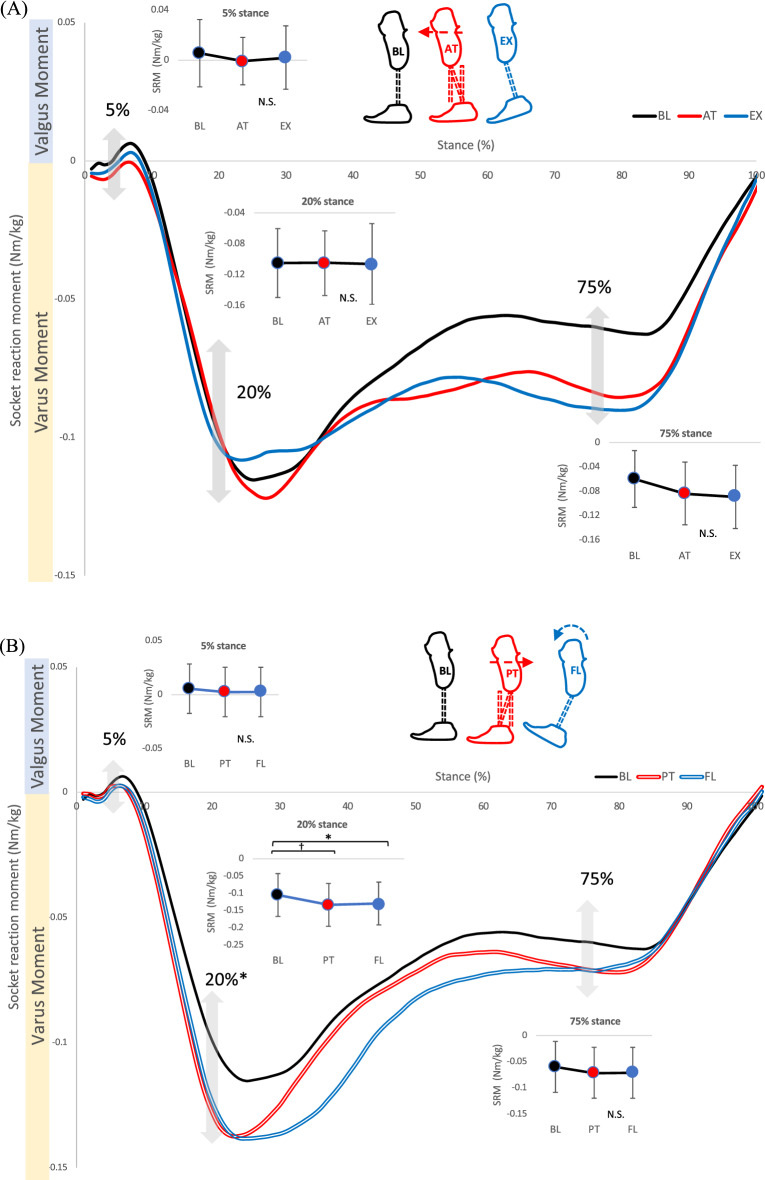
Socket reaction moment in the coronal plane under sagittal socket alignment changes (Comparison #1). (**A**) Anterior translation versus extension of the socket. Small line graphs indicate percent stance of socket reaction moments (5, 20, and 75%). (**B**) Posterior translation versus flexion of the socket. Small line graphs indicate percent stance of socket reaction moments (5, 20, and 75%). Whiskers indicate standard deviations. An asterisk (*) indicates *P* < 0.05. A dagger (†) indicates *P* < 0.01. Abbreviations: BL: baseline, AT: anterior translation, EX: extension, PT: posterior translation, FL: flexion, %stance: percent stance. N.S.: not significant.

There were no significant main effects on the walking speeds (*P* = 0.66: comparisons among baseline, anterior translation, and extension, and *P* = 0.90: comparisons among baseline, posterior translation, and flexion) (Table 2). There was a significant main effect at 20% stance among the baseline-posterior translation-flexion (*P* = 0.02). Post hoc tests indicated significant differences between the posterior translation and baseline (*P* = 0.007), as well as the flexion and baseline (*P* = 0.044, Table 3, Fig. 4A, B).

**Table 2:** Walking speed under each alignment condition.

**Alignment changes of the foot**				
	Plantarflexion	Baseline	Dorsiflexion	*P*-value & ES
Mean ± SD	1.07 ± 0.11	1.05 ± 0.15	1.06 ± 0.13	*P* = 0.67
95%CI	1.00–1.15	0.95–1.14	0.97–1.16	η_p_^2^ = 0.19
	Inversion	Baseline	Eversion	*P*-value & ES
Mean ± SD	1.07 ± 0.13	1.05 ± 0.15	1.07 ± 0.13	*P* = 0.76
95%CI	0.97–1.16	0.95–1.14	0.97–1.16	η_p_^2^ = 0.02
**Alignment change of the socket**				
	Anterior translation	Baseline	Extension	*P*-value & ES
Mean ± SD	1.05 ± 0.12	1.05 ± 0.15	1.07 ± 0.12	*P* = 0.66
95%CI	0.98–1.13	0.95–1.14	0.99–1.14	η_p_^2^ = 0.08
	Posterior translation	Baseline	Flexion	*P*-value & ES
Mean ± SD	1.06 ± 0.11	1.05 ± 0.15	1.05 ± 0.13	*P* = 0.90
95%CI	0.99–1.13	0.95–1.14	0.97–1.13	η_p_^2^ = 0.05
	Medial translation	Baseline	Adduction	*P*-value & ES
Mean ± SD	1.07 ± 0.11	1.05 ± 0.15	1.09 ± 0.13	*P* = 0.23
95%CI	1.00–1.14	0.95–1.14	1.01–1.17	η_p_^2^ = 0.20
	Lateral translation	Baseline	Abduction	*P*-value & ES
Mean ± SD	1.05 ± 0.11	1.05 ± 0.15	1.07 ± 0.12	*P* = 0.46
95%CI	0.98–1.12	0.95–1.14	1.00–1.15	η_p_^2^ = 0.24

*CI * confidence interval, *ES* effect size, SD standard deviation.

(η_p_^2^) indicates partial eta squared.

**Table 3:** Effects of sagittal alignment changes of the socket/foot on the coronal socket reaction moments (Comparison #1 & #2).

**Comparison #1**				
**5%stance SRM**				
	Anterior translation	Baseline	Extension	*P*-value & ES
Mean ± SD	0.00 ± 0.02	0.01 ± 0.03	0.00 ± 0.03	*P* = 0.41
95%CI	− 0.01 to 0.01	− 0.01 to 0.02	− 0.01 to 0.02	η_p_^2^ = 0.00
	Posterior translation	Baseline	Flexion	*P*-value & ES
Mean ± SD	0.00 ± 0.03	0.01 ± 0.03	0.00 ± 0.02	*P* = 0.81
95%CI	− 0.01 to 0.02	− 0.01 to 0.02	− 0.01 to 0.02	η_p_^2^ = 0.09
**20%stance SRM**				
	Anterior translation	Baseline	Extension	*P*-value & ES
Mean ± SD	− 0.11 ± 0.04	− 0.10 ± 0.04	− 0.11 ± 0.05	*P* = 0.74
95%CI	− 0.13 to -0.08	− 0.13 to -0.08	− 0.14 to -0.07	η_p_^2^ = 0.00
	Posterior translation	Baseline	Flexion	*P*-value & ES
Mean ± SD	− 0.13 ± 0.05	− 0.10 ± 0.04	− 0.13 ± 0.06	*P* = 0.02†a
95%CI	− 0.13 to − 0.09	− 0.13 to − 0.08	− 0.17 to -0.09	κ = 0.39
**75%stance SRM**				
	Anterior translation	Baseline	Extension	*P*-value & ES
Mean ± SD	− 0.08 ± 0.05	− 0.06 ± 0.05	− 0.09 ± 0.05	*P* = 0.05
95%CI	− 0.12 to -0.05	− 0.09 to -0.03	− 0.12 to − 0.06	η_p_^2^ = 0.64
	Posterior translation	Baseline	Flexion	*P*-value & ES
Mean ± SD	− 0.07 ± 0.05	− 0.06 ± 0.05	− 0.07 ± 0.05	*P* = 0.59
95%CI	− 0.09 to − 0.03	− 0.09 to − 0.03	− 0.10 to − 0.04	η_p_^2^ = 0.27
**Comparison #2**				
**5%stance SRM**				
	Plantarflexion	Baseline	Dorsiflexion	*P*-value & ES
Mean ± SD	− 0.01 ± 0.03	0.01 ± 0.03	0.00 ± 0.03	*P* = 0.49
95%CI	− 0.02 to 0.01	− 0.01 to 0.02	− 0.01 to 0.02	η_p_^2^ = 0.25
**20%stance SRM**				
	Plantarflexion	Baseline	Dorsiflexion	*P*-value & ES
Mean ± SD	− 0.12 ± 0.04	− 0.10 ± 0.04	− 0.11 ± 0.06	*P* = 0.27
95%CI	− 0.14 to − 0.10	− 0.13 to − 0.08	− 0.15 to − 0.07	η_p_^2^ = 0.16
**75%stance SRM**				
	Plantarflexion	Baseline	Dorsiflexion	*P*-value & ES
Mean ± SD	− 0.08 ± 0.05	− 0.06 ± 0.04	− 0.06 ± 0.05	*P* = 0.58
95%CI	− 0.11 to − 0.05	− 0.09 to − 0.03	− 0.09 to − 0.04	η_p_^2^ = 0.46

*CI* confidence interval, *ES* effect size, *SD* standard deviation, *SRM* socket reaction moment.

(†) indicates that Friedman tests were performed. (a) indicates results of post-hoc tests: Posterior translation versus Baseline (*p* = 0.007), Flexion versus Baseline (*p* = 0.044). (κ) indicates Kendall’s W. (η_p_^2^) indicates partial eta squared.

### Effects of sagittal alignment changes of the foot on the coronal socket reaction moments (comparison #2)

**Figure 5:**
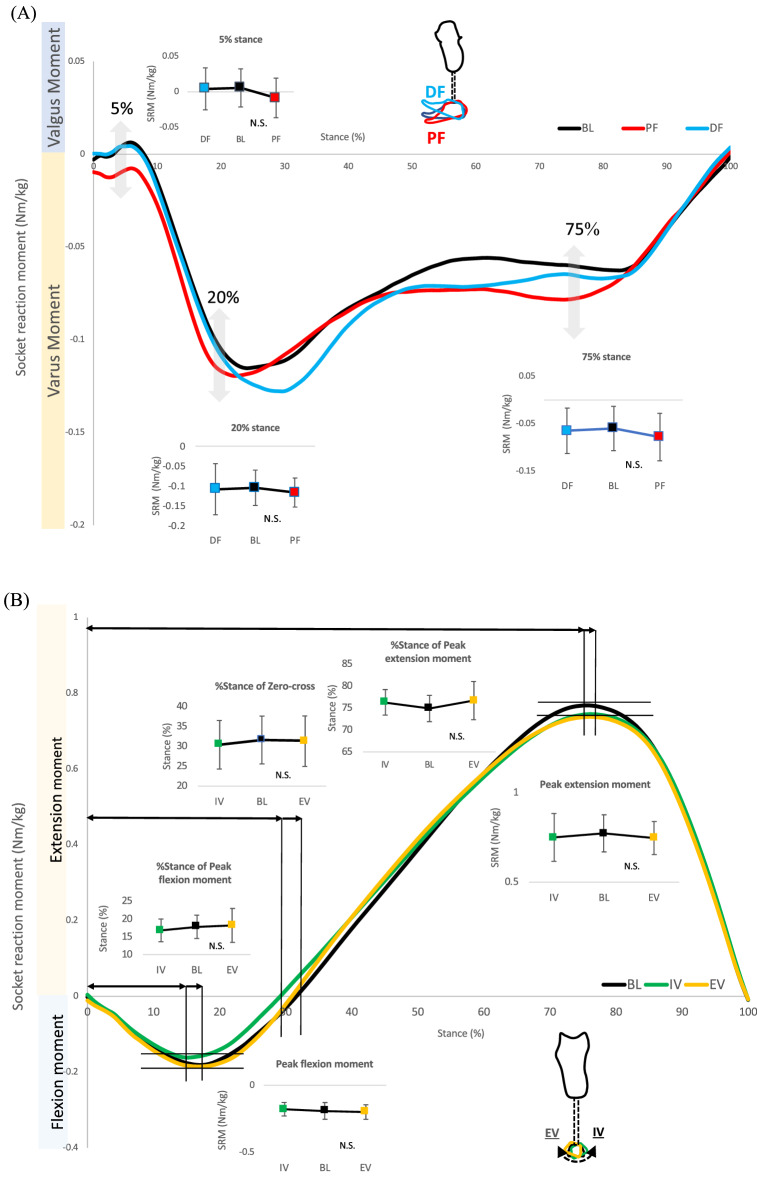
Socket reaction moment under foot alignment changes. (**A**) Socket reaction moment in the coronal plane under sagittal alignment changes of prosthetic feet (dorsiflexion vs. plantarflexion) (Comparison #2). Small line graphs indicate 5, 20, and 75 percent stance of socket reaction moments. Whiskers indicate standard deviations. (**B**) Socket reaction moment in the sagittal plane under coronal alignment changes of prosthetic feet (inversion vs. eversion) (Comparison #4). Small line graphs indicate peak flexion and extension socket reaction moments, percent stance of peak flexion and extension socket reaction moments and zero-cross. Whiskers indicate standard deviations. Abbreviations: BL: baseline, DF: dorsiflexion, PF: plantarflexion, IV: inversion, EV: eversion, N.S: not significant.

There were no significant main effects on walking speed (*P* = 0.67: comparisons among baseline, plantarflexion, and dorsiflexion) (Table 2). There were no significant main effects among the conditions in parameters related to magnitudes and timings of socket reaction moments (Table 3, Fig. 5A).

### Effects of coronal alignment changes of the socket on the sagittal socket reaction moments (comparison #3)

**Figure 6:**
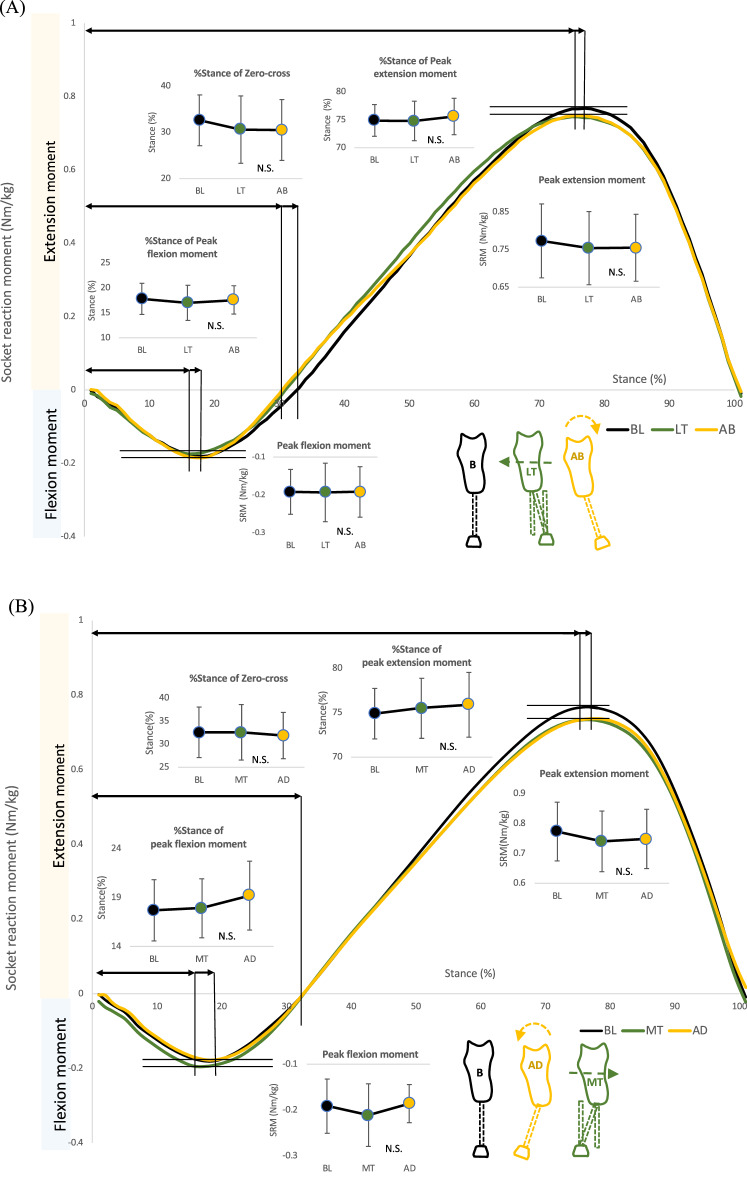
Socket reaction moment in the sagittal plane under coronal socket alignment changes (Comparison #3). (**A**) Abduction versus lateral translation of the socket. Small line graphs indicate peak flexion and extension socket reaction moments, Percent stance of peak flexion and extension socket reaction moments and zero-cross. (**B**) Adduction versus medial translation of the socket. Small line graphs indicate peak flexion and extension socket reaction moments, percent stance of peak flexion and extension socket reaction moments, and zero-cross. Whiskers indicate standard deviations. Abbreviations: BL: baseline, AB: abduction, AD: adduction, LT: lateral translation, MT: medial translation, %stance: percent stance, N.S.: not significant.

There were no significant main effects on walking speed (*P* = 0.46: comparisons among baseline, lateral translation, and abduction, and *P* = 0.23: comparisons among baseline, medial translation, and adduction) (Table 2). There were no significant main effects among the conditions in parameters related to magnitudes and timings of socket reaction moments (Table 4, Fig. 6A, B).

**Table 4:** Effects of coronal alignment changes of the socket on the sagittal socket reaction moments (Comparison #3).

**Comparison #3**				
**Peak extension moment**				
	Abduction	Baseline	Lateral translation	*P*-value & ES
Mean ± SD	0.75 ± 0.09	0.77 ± 0.10	0.75 ± 0.03	*P* = 0.39
95%CI	0.70 to 0.81	0.71 to 0.84	0.70 to 0.81	η_p_^2^ = 0.14
	Adduction	Baseline	Medial translation	*P*-value & ES
Mean ± SD	0.75 ± 0.10	0.77 ± 0.10	0.74 ± 0.01	*P* = 0.14
95%CI	0.69 to 0.81	0.71 to 0.84	0.68 to 0.80	η_p_^2^ = 0.50
**Peak flexion moment**				
	Abduction	Baseline	Lateral translation	*P*-value & ES
Mean ± SD	− 0.19 ± 0.07	− 0.19 ± 0.06	− 0.19 ± 0.08	*P* = 0.99
95%CI	− 0.23 to − 0.15	− 0.23 to − 0.15	− 0.24 to − 0.15	η_p_^2^ = 0.00
	Adduction	Baseline	Medial translation	*P*-value & ES
Mean ± SD	− 0.19 ± 0.04	− 0.19 ± 0.06	− 0.21 ± 0.07	*P* = 0.31
95%CI	− 0.23 to − 0.16	− 0.23 to − 0.15	− 0.25 to − 0.17	η_p_^2^ = 0.31
**Zero-cross**				
	Abduction	Baseline	Lateral translation	*P*-value & ES
Mean ± SD	30.50 ± 6.58	32.60 ± 5.78	30.60 ± 7.28	*P* = 0.20
95%CI	26.42 to 34.58	29.02 to 36.18	26.09 to 35.11	η^2^ = 0.28
	Adduction	Baseline	Medial translation	*P*-value & ES
Mean ± SD	31.90 ± 6.02	32.60 ± 5.78	32.60 ± 10.08	*P* = 0.82†
95%CI	28.78 to 35.02	29.02 to 36.18	28.87 to 36.33	κ = 0.02
**Percent stance of peak extension moment**				
	Abduction	Baseline	Lateral translation	*P*-value & ES
Mean ± SD	75.60 ± 3.26	75.30 ± 2.98	74.80 ± 3.54	*P* = 0.49
95%CI	73.58 to 77.62	73.45 to 77.15	72.60 to 77.00	η_p_^2^ = 0.16
	Adduction	Baseline	Medial translation	*P*-value & ES
Mean ± SD	75.90 ± 3.13	75.30 ± 2.98	75.50 ± 4.16	*P* = 0.34†
95%CI	73.64 to 78.16	73.45 to 77.15	73.40 to 77.60	κ = 0.01
**Percent stance of peak flexion moment**				
	Abduction	Baseline	Lateral translation	*P*-value & ES
Mean ± SD	17.60 ± 2.83	17.80 ± 3.29	17.00 ± 3.52	*P* = 0.67
95%CI	15.84 to 19.36	15.76 to 19.84	14.82 to 19.18	η_p_^2^ = 0.10
	Adduction	Baseline	Medial translation	*P*-value & ES
Mean ± SD	19.20 ± 3.51	17.80 ± 3.29	17.90 ± 3.01	*P* = 0.10
95%CI	17.02 to 21.38	15.76 to 19.84	16.03 to 19.77	η_p_^2^ = 0.50

*CI* confidence interval, *ES* effect size, *SD* standard deviation, *SRM* socket reaction moment.

(†) indicates that Friedman tests were performed. (κ) indicates Kendall’s W. (η_p_^2^) indicates partial eta squared.

### Effects of coronal alignment changes of the foot on the sagittal socket reaction moments (comparison #4)

There were no significant main effects on walking speed (*P* = 0.76: comparisons among baseline, inversion, and eversion) (Table 2). There were no significant main effects among the conditions in parameters related to magnitudes and timings of socket reaction moments (Table 5, Fig. 5B).

**Table 5:** Effects of coronal alignment changes of the foot on the sagittal socket reaction moments (Comparison #4).

**Comparison #4**				
**Peak extension moment**				
	Inversion	Baseline	Eversion	*P*-value & ES
Mean ± SD	0.75 ± 0.12	0.77 ± 0.10	0.75 ± 0.09	*P* = 0.21
95%CI	0.67 to 0.83	0.70 to 0.84	0.69 to 0.80	η_p_^2^ = 0.31
**Peak flexion moment**				
	Inversion	Baseline	Eversion	*P*-value & ES
Mean ± SD	− 0.18 ± 0.06	− 0.19 ± 0.06	− 0.20 ± 0.05	*P* = 0.31
95%CI	− 0.21 to − 0.15	− 0.23 to − 0.15	− 0.23 to − 0.17	η_p_^2^ = 0.42
**Zero-cross**				
	Inversion	Baseline	Eversion	*P*-value & ES
Mean ± SD	30.40 ± 5.78	32.60 ± 5.78	31.30 ± 6.02	*P* = 0.68
95%CI	26.82 to 33.98	29.02 to 36.18	27.57 to 35.03	η_p_^2^ = 0.13
**Percent stance of peak extension moment**				
	Inversion	Baseline	Eversion	*P*-value & ES
Mean ± SD	76.30 ± 2.76	75.30 ± 2.98	76.70 ± 4.12	*P* = 0.25
95%CI	74.59 to 78.01	73.45 to 77.15	74.14 to 79.26	η_p_^2^ = 0.35
**Percent stance of peak flexion moment**				
	Inversion	Baseline	Eversion	*P*-value & ES
Mean ± SD	16.80 ± 3.02	17.80 ± 3.29	18.20 ± 4.51	*P* = 0.25
95%CI	14.92 to 18.68	15.76 to 19.84	15.40 to 21.00	η_p_^2^ = 0.30

*CI* confidence interval, *ES* effect size, *SD* standard deviation, *SRM* socket reaction moment.

(η_p_^2^) indicates partial eta squared.

## Discussion

This study investigated the effects of alignment changes of the transtibial prosthetic socket (i.e., flexion/extension, abduction/adduction, and anterior/posterior, medial/lateral translation with equal displacement to counterpart angular alignment) and the foot (i.e., plantarflexion/dorsiflexion, inversion/eversion) on the out-of-plane socket reaction moments in order to determine the appropriate sequence of dynamic alignment. As walking speeds were not significantly different within each comparison group, alignment changes should be the only factor contributing to changes in the socket reaction moments.

The results showed that only the alignment changes of the socket in the sagittal plane (i.e., posterior translation and flexion) significantly increased the varus moment in the coronal plane. This was consistent with the previous study, which found that the extension of the socket significantly decreased varus moment in the coronal plane^32^. The alignment changes of the foot in the coronal and sagittal planes did not show significant changes in socket reaction moments in the out-of-planes (i.e., effects of sagittal alignment changes on coronal socket reaction moment, and effects of coronal alignment changes on sagittal socket reaction moment). This result was also in line with the previous study^32^. A prior study found that the alignment changes of the prosthetic foot in the transverse plane (i.e., toe-in and toe-out) significantly affected the coronal socket reaction moment^29^. This was the only significant effect of foot alignment changes on the out-of-plane moment.

The sagittal alignment of the socket affects the magnitude of flexion moment in the early stance when the ground reaction force is anterior to the knee joint^37^, and this may be a result of braking force originated from the residual limb and transferred to the socket to control knee extension. In the late stance, the alignment affects the extension moment to control knee flexion, as the ground reaction force vector during propulsion is directed behind the knee joint. When the transition of these forces are interrupted, the prosthesis may not move forward smoothly, leading to abnormal stress at the residuum-socket interface that likely affects the coronal moment. In contrast, the coronal alignment changes may not affect the sagittal socket reaction moment because six degrees of alignment changes might not be enough to influence braking and propulsion force. In clinical practice, angular adjustments greater than six degrees from the baseline alignment may not be adopted because it approaches the limit of angular changes for the components designed for daily use. Further study is needed to investigate the relationship between the sagittal alignment and coronal moment in a systematic and comprehensive way.

Previous studies revealed systematic effects of sagittal alignment changes of both the socket and the foot on sagittal socket reaction moments, and the effects of coronal alignment changes of the socket and the foot on coronal socket reaction moments^27,38,39^. It is likely that the sagittal socket reaction moment is affected merely by sagittal alignment changes of both the socket and the foot, whereas the coronal socket reaction moment is affected by alignment changes in all three planes: coronal alignment changes of both the socket and the foot, sagittal alignment changes of the socket, and transverse alignment changes of the foot. Therefore, it is suggested to first determine the sagittal alignment, because even if coronal or transverse alignment changes are performed after sagittal alignment is completed, these changes may not significantly affect the sagittal socket reaction moments. Although sagittal alignment changes may potentially influence the coronal socket reaction moment, the coronal moment can be tweaked by alignment changes in other planes (i.e., coronal and transverse) during dynamic alignment.

To establish coronal alignment, it might be necessary to consider the effects of transverse alignment. It is recommended that toe-in and toe-out angles should be determined according to the intact side at static alignment^12^. This could be mainly due to the aesthetic preference of the users. Therefore, toe-in/toe-out angles are generally determined at static alignment. However, the effect of transverse alignment determined during static alignment should also be considered in dynamic alignment because the transverse alignment changes systematically affect the coronal socket reaction moment^29^. For example, when the prosthetic foot is internally rotated (i.e., toe-in) at dynamic alignment, it may increase the magnitude of varus moment in the late stance during gait when compared to bench alignment^29^. Greater varus moment can also be induced by excessive coronal alignment changes (e.g., eversion of the foot^39^ or lateral translation/abduction of the socket^23,27,38^). Thus, in this case, there should be three options for alignment adjustments in transverse and/or coronal planes (i.e., decrease in toe-in angle, decrease in eversion, and decrease in lateral translation) and further adjustment of sagittal alignment may not be necessary.

There was no significant difference related to the timing of the socket reaction moment in the out-of-plane (i.e., effects of sagittal alignment changes on the timing of the coronal socket reaction moment, and effects of coronal alignment changes on the timing of the sagittal socket reaction moment). Our previous study suggested that in the sagittal plane angular changes mainly affect the timing of the socket reaction moment and translational changes affect primarily the magnitude of the socket reaction moment^33^. Also, this study suggested that translational changes in the coronal plane affect the magnitude of socket reaction moment primarily in the mid-to-late stance and angular changes show a similar effect, but to a lesser degree in the late stance^29^. Furthermore, it should be noted that the effects of angular changes in the socket are equivalent to the combined effects of angular and translational changes in the foot (Fig. 1)^33,39^. Therefore, whether an angular or translational adjustment should be performed first should be determined based on their effect on timing and/or magnitude of socket reaction moment.

This study has some limitations. As male participants were predominant, it is hard to further elucidate the possible effect of gender. As the length of residual limb and leg length varied among participants, the same amount of angular change could introduce different displacements (*d* in Fig. 1) at the distal end. Participants in the study have relatively high activity levels (K3-4) and it remains unclear if similar findings could be revealed in populations with low activity levels (Table 1). The prosthetic foot was controlled in this study and the effects of different prosthetic foot design remain unclear. The acclimation period was also brief so the long-term effects are unknown.

**Figure 7:**
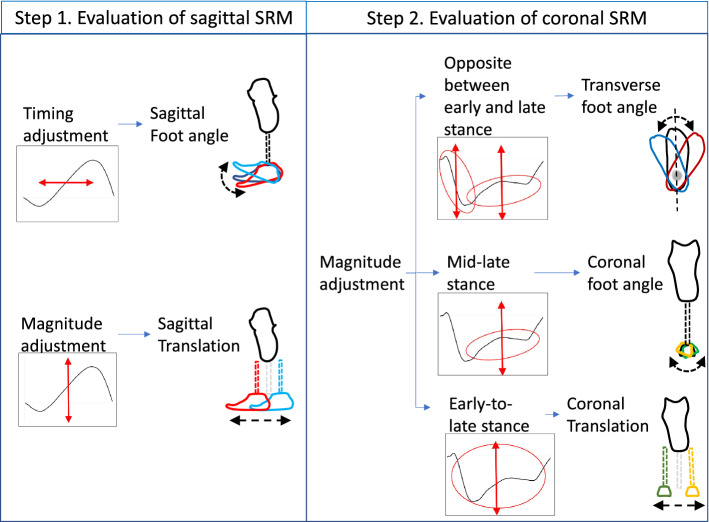
A flowchart of dynamic alignment using the socket reaction moment. Abbreviation: SRM: socket reaction moment.

In conclusion, this study showed that sagittal alignment may affect both the sagittal and coronal socket reaction moments, and coronal alignment may affect only the coronal socket reaction moment. According to these findings, the appropriate sequence of adjusting the alignment of transtibial prostheses should be first in the transverse plane in static alignment (i.e., adjustment of toe-in/toe-out angles for standing symmetry)^29^, followed by dynamic alignment (i.e., adjustment of toe-in/toe-out angles for gait symmetry), then in the sagittal alignment in dynamic alignment procedure, and lastly in the coronal plane (Fig. 7). It should be noted that the coronal socket reaction moment may be affected by alignment changes in all three planes. Therefore, our hypothesis is supported, and the adjustment of coronal alignment should be performed lastly considering the out-of-plane effects. These findings could serve as a clinical guideline for alignment processes in transtibial prostheses. However, future studies with a larger sample size, more representative population with a wider selection of prosthetic feet and socket designs is needed to validate this guideline and implementation in clinical practice.

## Data Availability

The datasets generated for this study are available from the corresponding author on reasonable request.
